# Complete mitochondrial genome of the common Cuora (*Cuora mccordi*), China

**DOI:** 10.1080/23802359.2022.2095938

**Published:** 2022-07-25

**Authors:** Hewei Jiang, Yawei Shen, Hao Wang, Hailong Zhao, Xiaowu Chen

**Affiliations:** a Key Laboratory of Exploration and Utilization of Aquatic Genetic Resources, Ministry of Education, Shanghai Ocean University, Shanghai, China; b Beijing Guidao Biology Science and Technology Co., Ltd., Beijing, China; c Shanghai Collaborative Innovation for Aquatic Animal Genetics and Breeding, Shanghai Ocean University, Shanghai, China

**Keywords:** Complete mitochondrial genome, *Cuora mccordi*, *Cuora*

## Abstract

We report the complete mitochondrial genome of *Cuora mccordi*. The complete genome is a closed circular molecule of 16,551 bp, with an overall base composition of 34.06% for A, 26.73% for T, 12.84% for G, and 26.37% for C. The A + T content is 60.79%. The full length consists of 13 protein-coding genes, 22 tRNA genes, two rRNA genes, and one control region (D-loop). Phylogenetic analysis results showed that the mitogenome of *Cuora mccordi* was the closest to *Cuora pani*. The complete mitochondrial genome of *Cuora mccordi* (GenBank accession number: OM327796) can aid in understanding evolutionary relationships within *Cuora*.

*Cuora mccordi*, a kind of unique *Cuora* to China, is only distributed in Guangxi region of China. The species was first discovered by Ernst in 1988 (Zhou et al. 2008). The head and neck of *Cuora mccordi* is yellow to orange, the back of the head is green, and an orange postorbital stripe with a black border. The dorsal carapace is raised and reddish brown, and the abdominal carapace is yellow with a black spot that almost covers most of the abdomen (Zhou et al. 2008). Due to the on-going habitat destruction on Guangxi, China, the current population of this *Cuora* is declining (Tang et al. 2019). *Cuora mccordi* was reclassified from Least Concern to Vulnerable IUCN status in 2000. The IUCN Red List of Threatened Species (https://www.iucnredlist.org/search?query=Cuora%20mccordi&searchType=species) defined *Cuora mccordi* as critically endangered (CR).

The samples used in this study were obtained from individuals cryopreserved after natural death in October 2021 at Beijing Guidao Biology Science and Technology Co., Ltd. (116.318076E, 40.0901N), China. They were transported to and stored in the College of Fisheries and Life Science, Shanghai Ocean University with voucher number: SOUM202110 (Ms. Jiang, jiaghewei1@163.com). All procedures were strictly in accordance with the ‘Guidelines for the Handling and Use of Laboratory Animals at Shanghai Ocean University’. The Animal Ethics Committee of Shanghai Ocean University approved the research protocol. DNA sequencing (paired-end sequencing) was performed in this study using the MGISEQ-2000 platform. The mitotic genome sequence was annotated by MITOS (Bernt et al. 2013) and the phylogenetic tree was structured by MEGA 7.

The complete mitochondrial genome of *Cuora mccordi* is 16,551 bp and consists of 37 genes, including 13 protein-coding genes (PCGs), two ribosomal RNA genes (12S ribosomal RNA and 16S ribosomal RNA), 22 tRNA genes, and one control region (D-loop). Overall base composition is 34.06% A, 26.73% T, 12.84% G, and 26.37% C, with A + T content of 60.79%. The total length of the 13 PCGs is 11,378 bp; other than ND6, which is in the heavy strand (H strand), all of these genes are encoded on the same strand. Among the 13 PCGs, the start codons are GTG in COX1, CCT in ND6, and ATG in the remaining 11 PCGs (ND1, ND2, ND3, ND4, ND4L, ND5, ATP6, ATP8, CYTB, COX2, and COX3). The total length of the 22 tRNA genes is 1555 bp, with genes ranging from 1 to 70 bp interspersed along the whole genome. The sequence lengths of the 12S RNA gene, the 16S RNA gene, and the D-loop region (control region) are 962, 1602, and 1079 bp, respectively. All of this information and the assembled sequences were submitted to GenBank under accession number OM327796. In addition, the raw sequencing data were deposited in SRA (SRA no. PRJNA824477).

To verify the phylogenetic position of *Cuora mccordi*, a phylogenetic analysis was carried out based on the complete mitogenomes of other turtles in the family. Sequence dataset was aligned using ClustalW and analyzed using the neighbour-joining method and the Tamura-Nei model (Tamura and Nei 1993) in MEGA 7.0 (Kumar et al. 2016). The examined taxa were represented by the bootstrap consensus tree generated from 1000 repetitions (Felsenstein 1985). The phylogenetic tree showed that *Cuora pani* is the closest phylogenetic relationship with *Cuora mccordi* (Figure 1). The genome information gotten in this might contribute to future studies on the molecular evolution and phylogeny of *Cuora*.

**Figure 1.**
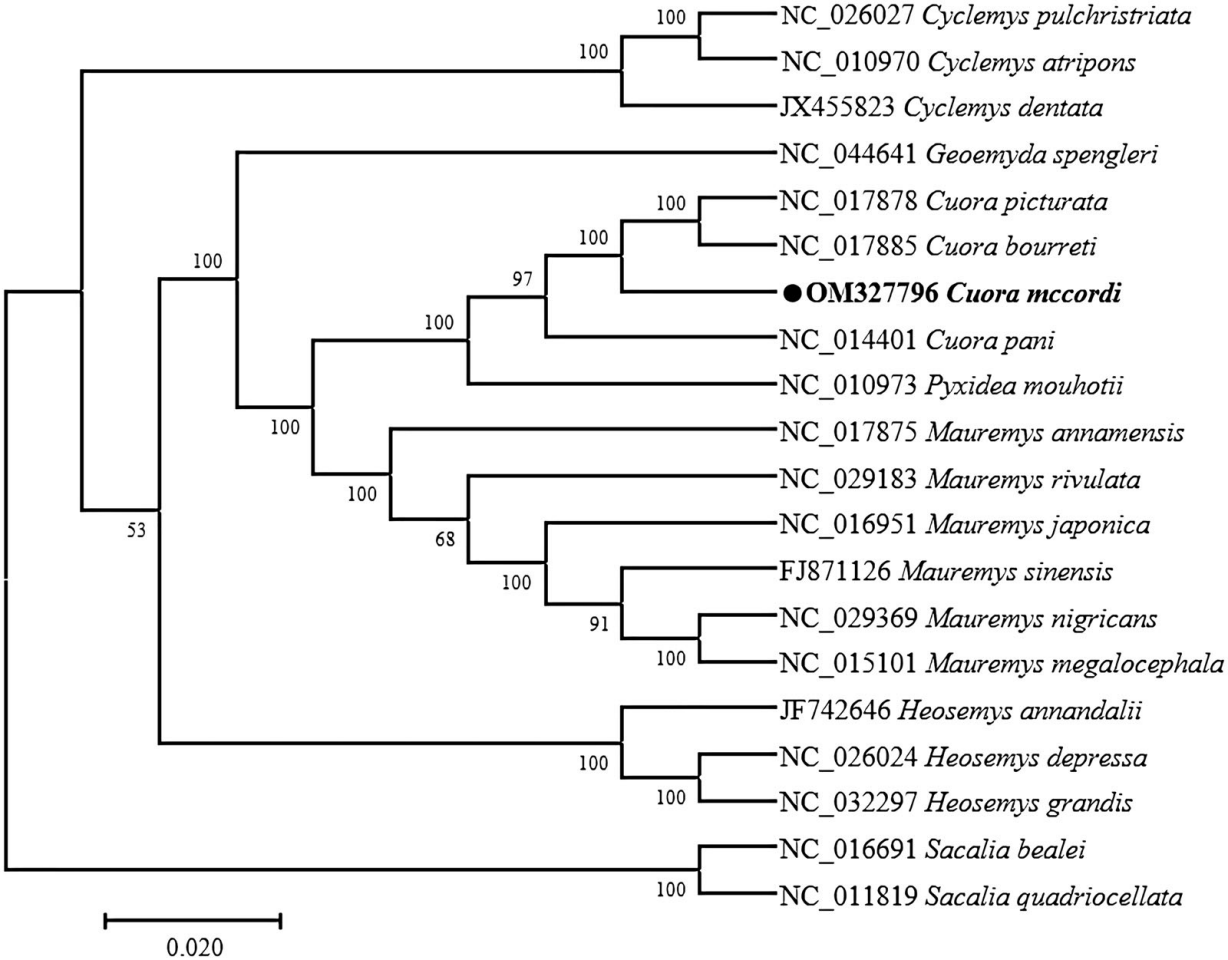
Phylogenetic tree generated using the maximum-likelihood method based on complete mitochondrial genomes of 20 species.

## Data Availability

The data that support the findings of this study are openly available in GenBank of NCBI at https://www.ncbi.nlm.nih.gov/, reference number OM327796. The associated Bio-Project, SRA, and Bio-Sample numbers are PRJNA824477, SRR18675450, and SAMN27404607, respectively.
